# Health supervision for children and adolescents with 16p11.2 deletion syndrome

**DOI:** 10.1101/mcs.a006316

**Published:** 2023-12

**Authors:** Wendy K. Chung, Faranak F. Herrera, Simon's Searchlight Foundation

**Affiliations:** 1 Harvard Medical School and Boston Children's Hospital, Boston, Massachusetts 02115, USA;; 2 16p Genetic Foundation, Irvine, California 92603, USA;; 3 New York, New York 10010, USA

**Keywords:** abdominal obesity, abnormal speech discrimination, apraxia, autism, delayed fine motor development, delayed gross motor development, dysgraphia, dyslexia, extrapyramidal dyskinesia, grammar-specific speech disorder, intellectual disability, mild, intellectual disability, moderate, language impairment, moderate expressive language delay, moderate receptive language delay, narrow palate, poor fine motor coordination, poor gross motor coordination, precocious puberty in males, sleep disturbance

## Abstract

Rare genetic conditions are challenging for the primary care provider to manage without proper guidelines. This clinical review is designed to assist the pediatrician, family physician, or internist in the primary care setting to manage the complexities of 16p11.2 deletion syndrome. A multidisciplinary medical home with the primary care provider leading the care and armed with up-to-date guidelines will prove most helpful to the rare genetic patient population. A special focus on technology to fill gaps in deficits, review of case studies on novel medical treatments, and involvement with the educational system for advocacy with an emphasis on celebrating diversity will serve the rare genetic syndrome population well.

This clinical review is designed to assist the pediatrician, family physician, or internist in caring for the child, adolescent, adult, and family in whom a diagnosis of 16p11.2 deletion syndrome has been confirmed by chromosome analysis. Although a pediatrician's initial contact with the child is usually within the first 5 years of life, occasionally an adolescent or adult will be tested and diagnosed after their sibling or child has been diagnosed with the same condition. Age-specific guidance for the clinician is provided in Supplemental Figure 1. 16p11.2 deletion is the most common genetic cause of neurodevelopmental disorders (Männik et al. 2015) and autism spectrum disorder (ASD) and is characterized by motor speech disorder, language disorder, motor coordination difficulties, some degree of developmental delay, below-average cognition, learning disabilities in both verbal and nonverbal domains, and psychiatric conditions (Table 1; Miller et al. 1993). Although those with 16p11.2 deletion have some degree of development delay, the severity can vary significantly. Awareness and prompt attention to the issues are important in optimizing lifelong outcomes. There are no pathognomonic phenotypic features that can facilitate rapid clinical diagnosis; however, vertebral anomalies (often leading to scoliosis) (Zufferey et al. 2012), hearing impairment, cardiac malformations (Zufferey et al. 2012), congenital anomalies of the kidneys and urinary tract (Haller et al. 2018), slightly below-average height (Zufferey et al. 2012), macrocephaly (Zufferey et al. 2012), and craniosynostosis (Zufferey et al. 2012) are noted in some individuals with the deletion. No patient will have all of these features.

**Table 1.** Medical problems common in 16p11.2 deletion syndrome

Condition	%
Developmental delay	100
Psychiatric/behavioral issues	90
Motor speech disorders	80
Language disorder	80–90
Obesity	75
Motor coordination difficulties	60
Autistic features/autism	25
Seizures	25
Vertebral anomalies	21
Hearing impairment	<11
Paroxysmal kinesigenic dyskinesia	<9
Cardiac malformations	6

16p11.2 recurrent deletion should be considered in individuals with

- motor speech disorders (Fedorenko et al. 2016; Demopoulos et al. 2018),
- language disorders (Mei et al. 2018; Jiménez-Romero et al. 2022),
- learning difficulties/intellectual disability (Miller et al. 1993; Hanson et al. 2015; Anguera et al. 2016),
- social impairments with or without ASD (Miller et al. 1993; Zufferey et al. 2012; Grove et al. 2019),
- macrocephaly (Miller et al. 1993),
- Chiari I malformation/cerebellar tonsillar ectopia (Miller et al. 1993),
- seizures/epilepsy (Moufawad El Achkar et al. 2022),
- vertebral anomalies (Miller et al. 1993),
- obesity in the setting of developmental delay (Bachmann-Gagescu et al. 2010; Perrone et al. 2010; Zufferey et al. 2012),
- breath holding (Bhat et al. 2018).

The BP4–BP5 16p11.2 recurrent deletion is often caused by a de novo deletion. The 16p11.2 BP4–BP5 deletion diagnosis is established with the identification of a heterozygous −593-kb deletion at Chromosome 16 (nucleotides 29,638,676–30,188,531 in the reference genome GRCh38). The diagnosis can be made by chromosome microarray or exome/genome sequencing, or with targeted quantitative polymerase chain reaction (qPCR) testing or fluorescence in situ hybridization (FISH). However, not all deletions are de novo, and autosomal dominant transmission affecting both males and females in multiple generations is also observed. Testing should be offered to parents of the first person diagnosed in the family. Should either parent have the same deletion, all children of that parent should also be tested. Prognostication from prenatal testing is challenging given the inherent difficulty in accurately predicting the phenotype.

Formal counseling by a clinical geneticist or genetic counselor is recommended for all families after diagnosis. The bulk of the medical attention required for proper treatment of this patient population has historically fallen onto the family of the patient, leading to heterogeneous and inconsistent treatment plans. The transition of the medical management to shift to the primary care setting should provide the best medical home.

Several areas require ongoing assessment throughout childhood and should be reviewed at every health supervision visit and at least annually. These areas include

- support available to family,
- participation in a family-centered medical home,
- age-specific 16p11.2 deletion syndrome medical and developmental surveillance,
- appropriate neurocognitive evaluation with early and aggressive therapy to target any deficits,
- early (starting in toddler years) monitoring of nutrition and activity levels to maintain appropriate weight,
- financial and medical support programs and long-term financial planning for which the child and family may be eligible,
- injury and abuse prevention.

## HEALTH SUPERVISION FROM BIRTH TO 1 MO: NEWBORN INFANTS

It is currently not common that a newborn infant with 16p11.2 microdeletion syndrome is diagnosed at or prior to birth. Research studies are under way considering the addition of certain genetic variants to prenatal and/or newborn screening that may lead to earlier diagnosis. Congenital heart disease and/or congenital diaphragmatic hernia are occasionally identified on ultrasound, leading to a prenatal diagnosis (Prenatal Diagnosis: Vol 38, No 6; Genesio et al. 2018; Liu et al. 2023). Newborns with congenital anomalies typically have a chromosome microarray that will establish the diagnosis. In such cases, it is important that all other 16p11.2 deletion manifestations are screened and addressed after birth.

### Examination

The initial steps in evaluating a newborn infant for 16p11.2 deletion are a careful review of the family history and prenatal information.

This should include:

- results of prenatal genetic testing and fetal ultrasound, if performed;
- family history of previous children born with 16p11.2 recurrent deletion, ASD, developmental delay, and/or early childhood obesity.

For children who have had the diagnosis made prenatally, a copy of the genomic testing should be obtained and added to the child's medical record.

### Evaluate For

- feeding problems (feeding difficulties can include gastroesophageal reflux, dysphagia, micrognathia, and hypotonia with poor tongue control; speech evaluation is advised),
- hearing loss,
- vertebral anomalies,
- cardiac malformations (perform an echocardiogram),
- signs and symptoms of neuroblastoma at each well visit (although quite rare, 16p11.2 deletion predisposes children to neuroblastoma) (Egolf et al. 2019).

### Anticipatory Guidance Given between Birth and 1 Mo of Age

- Discuss signs suggestive of seizure activity and how the family may seek help.
- Discuss symptoms related to sleep disorders such as snoring and witnessed apnea (Kamara et al. 2023).
- Discuss expectations for early intervention and the availability of early intervention services and therapies in the community. Initiate referral for physical therapy, unless medically contraindicated.

## HEALTH SUPERVISION FROM 1 MO TO 1 YR: INFANCY

### Physical Examination and Laboratory Studies

- History and physical exam.
- Monitor weight every 6 mo.
- Head circumference should be checked at every visit because of the association between macrocephaly and obesity with 16p11.2 deletion (Loviglio et al. 2017).
- Review feeding. Consider consultation for proper nursing. Speech and language pathology evaluation for appropriate orofacial muscle tone is recommended by age 12 mo, especially if there are any signs of micrognathia (Atli et al. 2022).
- Review prior hearing evaluations. If the infant has passed the newborn screening, rescreen at 6 mo and then annually (Berman et al. 2016).
- Should the child present with recurrent upper respiratory infections, obtain a complete blood count with differential and immunoglobulin levels at any age and by age 5 as a point of reference to assess for potential immunologic anomalies (Shiow et al. 2009; Giannuzzi et al. 2022).
- Rule out anemia and iron deficiency, including a complete blood count with differential, ferritin, and total iron-binding capacity beginning at 1 yr of age and annually thereafter. Iron deficiency is also associated with breath-holding syndrome (Bhat et al. 2018) and sleep difficulties.
- Obtain an electroencephalogram if there is any sign of seizures (Steinman et al. 2016).
- Any recurring movements should initiate referral to neurology for possible paroxysmal kinesigenic disorder (Wang et al. 2011).
- Assess for congenital scoliosis (Wu et al. 2015).

### Anticipatory Guidance from 1 Mo to 1 Yr

- Review the availability of resources, including support groups and organizations that help with the navigation of community and financial resources, at least once in the first year of life (Bamonte 2015) and then at every health maintenance visit.
- Assess the emotional status of caregivers and intrafamilial relationships at each health maintenance visit. Inquire about how siblings are adjusting to the new baby and offer education to support the siblings as needed (Akid 2022; Akid and Lara 2022).
- Review expected developmental trajectories and the importance of early intervention at each child visit (Bernier et al. 2017).

## HEALTH SUPERVISION FROM 1 TO 5 YR: EARLY CHILDHOOD

### Physical Exam and Specialty Collaboration

- History and physical exam.
- It is recommended that neurocognitive and neuropsychiatric testing be done at diagnosis and monitored annually to adjust therapies. Lower than actual intelligence quotient (IQ) can be seen early on. Explain that the child's IQ, if the inheritance is de novo, should not be more than two standards of deviation from that of the family's average IQ (Moreno-De-Luca et al. 2015).
- Evaluation and prompt treatment for speech and language disorders, including expressiveness, receptiveness, apraxia (Mei et al. 2018), auditory feedback, and motor control (Demopoulos et al. 2018), as well as social pragmatic communication disorders (Jiménez-Romero et al. 2022). One or all of these areas of speech can be affected.
- Monitor weight and body mass index (BMI). Obesity can begin in the toddler years (Miller et al. 1993). Altered satiety presents prior to the onset of obesity (Maillard et al. 2016), serving as a flag to control portion size. Referral to a nutritionist is indicated prior to a significant increase in BMI *Z*-scores (Faccioli et al. 2023).
- Monitor for any sensory-seeking behavior and refer for neurobehavioral therapy (Osório et al. 2021).
- Monitor for hypotonia and coordination disorders (Hinkley et al. 2019) with evaluation by occupational and physical therapy to address tone and coordination abnormalities. Early signs may manifest in difficulty with fine motor tasks.
- Evaluation for auditory-processing challenges by age 3 (Jenkins et al. 2016) followed by appropriate treatment is recommended. Given the potential for improvement when therapy is started at an early age (Bernier et al. 2017), evaluation by age 3 is recommended despite traditional recommendations to wait until age 7 to assess for auditory-processing difficulties.
- Evaluation for visual-processing disorders (Choi et al. 2023) is recommended (Kooiker et al. 2020) by age 4 with an experienced developmental optometrist and subsequent vision therapy if necessary.
- Brain magnetic resonance imaging (MRI) to assess for Chiari malformation if there are recurring headaches (Schaaf et al. 2011).
- Obtain an electroencephalogram with any symptoms suggestive of seizures (Moufawad El Achkar et al. 2022).
- Symptoms of repetitive movements should prompt follow-up with neurology for possible paroxysmal kinesigenic dyskinesia (Termsarasab et al. 2014).
- Discuss symptoms related to sleep disorders such as snoring, witnessed apnea, daytime somnolence, and attention deficit at every visit (Kamara et al. 2023).

#### Anticipatory Guidance from 1 to 5 Yr

- Review early intervention including speech therapy, occupational therapy, physical therapy, and applied behavioral analysis at all visits (Bernier et al. 2017).
- Discuss the transition from early intervention to preschool that should occur at age 3. Emphasize the importance of obtaining a professional advocate and an educational specialist to navigate services needed through the school system (Rehm et al. 2013) in obtaining an Individualized Educational Plan (IEP). The IEP is a legal document that should take the scientific findings of all recommended neuropsychiatric evaluations, including parental concerns, and translate them into actionable goals to address any deficits found while capitalizing on the child's strengths. This translation is easily missed without an educational specialist or an advocate well versed in both education sciences and the law of the state the child resides in.
- Review the child's behavior to support the family dynamics and siblings and educate on parent–child interaction therapy (Campbell et al. 2023).
- Provide resources to help navigate community and financial resources including child care.
- Encourage healthy diet and physical activity for the prevention of obesity.
- Discuss probiotics with *Lactobacillus reuteri* (Buffington et al. 2016) and *Enterococcus faecalis* (Buffington et al. 2016).

## HEALTH SUPERVISION FROM 5 TO 12 YR: LATE CHILDHOOD

### Physical Exam and Specialty Collaboration

- History and physical exam.
- Neurocognitive and neuropsychiatric testing to monitor progress at diagnosis, repeated annually.
- Evaluation and prompt treatment for speech and language disorders including expressiveness, receptiveness, apraxia (Mei et al. 2018), auditory feedback, and motor control (Demopoulos et al. 2018) as well as social pragmatic communication disorders (Jiménez-Romero et al. 2022). One or all of these areas of speech can be affected.
- Monitor weight and BMI at every visit. Provide family with dietary and exercise guidance and offer nutrition follow-up to prevent and *treat* obesity (Faccioli et al. 2023).
- Screen for attention-deficit/hyperactivity disorder (ADHD) by age 5 (Niarchou et al. 2019).
- Should ADHD be diagnosed, consider stimulants, which may help with attention deficit, speech (Rausch et al. 2017), motor coordination, and social reciprocity. Follow up with psychiatry experienced with genetic disorders.
- Should a child carry the diagnosis of autism spectrum disorder (ASD) or other social impairments, follow up with applied behavioral analysis and social skills classes (Eckes et al. 2023).
- Monitor behavioral issues that may interfere with gaining independence, such as attention, rigidity in thought, and difficulty with communication and transitions.
- Behavioral evaluation by a board-certified behavioral analyst between ages 5 and 7.
- Educationally based mental health services assessment should be completed between the ages of 5 and 7, with reassessment as part of the IEP.
- Evaluation for auditory- and visual-processing disorder if not done already and under continued specialty care (Jenkins et al. 2016; Choi et al. 2023).
- Obtain an electroencephalogram with any symptoms suggestive of seizures (Moufawad El Achkar et al. 2022).
- Monitor for hypotonia and coordination challenges with appropriate follow-up in physical and occupational therapies (Steinman et al. 2016).
- Discuss symptoms related to sleep disorders such as snoring, witnessed apnea, daytime somnolence, and attention issues at every visit (Kamara et al. 2023) and treat accordingly.
- Symptoms of repetitive movements should prompt follow-up with neurology for possible paroxysmal kinesigenic dyskinesia (Termsarasab et al. 2014).
- If speech does not improve with therapy, consider evaluation by an otolaryngologist for possible surgical interventions (adeno–tonsillectomy, frenectomy) or orthodontic measures (maxillary palate expansion) to correct anatomic impediments of speech. Orthodontist evaluation by age 5.

#### Anticipatory Guidance from 5 to 12 Yr

- Review the child's development and appropriateness for transitions to elementary or middle school. Advise that the family obtain a professional advocate to be sure that the child's individual educational plan goals reflect the needs identified in the neurocognitive, psychiatric, and behavioral evaluations.
- Discuss the importance of appropriate physical activity and organized sports despite possible impediments with hypotonia and lack of coordination.
- Encourage caregivers to promote self-reliance skills with developmentally appropriate responsibilities.
- Encourage reliance on technology such as speech-to-text for those with dysgraphia.
- Advocate for treatment and accommodations for dyslexia.
- Counsel on the transition through puberty.

## HEALTH SUPERVISION FROM 12 TO 21 YR OR OLDER: ADOLESCENCE TO EARLY ADULTHOOD

### Physical Exam and Specialty Collaboration

- History and physical exam.
- Monitor weight and BMI at every visit. Provide family with dietary and exercise guidance as well as offering nutrition follow-up to prevent and *treat* obesity (Faccioli et al. 2023).
- Obtain a basic metabolic panel, fasting lipids, and a hemoglobin A1C annually if the patient has a BMI > 95%. Manage metabolic conditions.
- Monitor speech, physical functionality, and cognitive development annually or sooner if indicated or after a certain treatment regimen to monitor efficacy.
- Obtain an electroencephalogram with any symptoms suggestive of seizures (Moufawad El Achkar et al. 2022).
- Discuss symptoms related to sleep disorders such as snoring, witnessed apnea, daytime somnolence, and attention issues at every visit (Kamara et al. 2023) and treat accordingly.
- Girls and women with autism are often underdiagnosed (Hull et al. 2020). Low threshold for neurocognitive and neuropsychiatric re-evaluation if indicated.
- Although very rare, any decline in cognitive performance between the ages of 13 and 18 should trigger re-evaluation with mental health to monitor for psychosis (MacCabe et al. 2013). Discuss the importance of reducing major life stressors such as a move during this delicate developmental period.

#### Anticipatory Guidance from 12 to 21 Yr and Older

- Discuss the importance of appropriate physical activity and organized sports despite possible impediments with hypotonia and lack of coordination.
- Discuss long-term financial planning with the patient and family from early adolescence.
- Discuss the appropriateness of school placement, vocational training, and postsecondary education within the school or community setting.
- Monitor and encourage independence with hygiene and self-care. Provide guidance and resources on healthy sexual development.
- Follow age-appropriate guidelines for evidence-based health and cancer screening.
- Provide guidance on the transition from pediatric/adolescent care to an adult medical home model (White et al. 2018).
- Discuss the possibility of an earlier age at menarche for girls and a younger than average age at the onset of pubertal traits for boys (Männik et al. 2019).
- Discuss the importance of conservatorship with the patient's caregivers before the age of 18 if functional status dictates.
- Discuss safe sex practices and reproductive guidance including contraception with adolescents and adults. Referral to genetic counseling for reproductive advice is advised. 16p11.2 deletion is transmitted from parent to child in an autosomal dominant manner (Miller et al. 1993).

## FUTURE CONSIDERATIONS

Personalized care and increased diagnosis of genetic syndromes is a fairly new and ever-evolving field. While caring for a patient and their family with 16p11.2 deletion syndrome, the focus of any primary care provider will need to be diligent management of syndrome manifestations along with continually staying up to date on changes to guidelines. A focus on using technological advances for improved functionality as well as ongoing emphasis on pharmacological and nutritional management will serve this patient population well. Managing family expectations on prognosis may be challenging. Given a global decrease in processing speed, not every area of deficit will be optimized. However, a focus on functionality and independence is a helpful overall strategy.

## RESOURCES FOR FAMILIES

### Prenatal and Infancy

- Letter Case: https://www.lettercase.org. Provides prenatal and postnatal counseling for families.
- March of Dimes: http://www.marchofdimes.com. Information for parents on health issues related to pregnancy and birth defects.
- Probably Genetic: https://www.probablygenetic.com. Offers free and at-home genetic testing for eligible patients.

### Childhood

- 16p11.2 Deletion Foundation: https://16pdel.org. Resource site for patients and families of those with 16p11.2 deletion syndrome with up-to-date information, education, potential therapies, and caregiver support.
- Akid N. *What are my genes: curious kid series*. California: Amazon; 2022.
- Akid N, Lara A. *What is variation: curious kid series*. California: Amazon; 2022.
- Akid N. *Just breath!: curious kid series, a family's Kintsugi story of 16p11.2 microdeletion syndrome*. California: Amazon; 2024.
- PCIT: https://www.parentchildinteractiontherapy.com. An evidence-based treatment program designed for caregivers and their young children (2–7 yr old) who are experiencing social, behavioral, and/or emotional difficulties.
- Project ImPACT: https://www.project-impact.org. Project ImPACT is a coaching program for parents of young children with autism and related social communication delays. Project ImPACT teaches parents strategies they can use to help their child develop social, communication, imitation, and play skills during daily routines and activities.
- Undivided: https://www.undivided.io. Assist parents to have more time, more money, and more emotional bandwidth to navigate medical and educational systems and find their place within a supportive community.
- The Mighty Corporation: https://corp.themighty.com. Community of people with lived experience sharing their honest stories. They help people connect with others around mental health, chronic illness, rare diseases, and disability.
- Angel Sense: https://www.angelsense.com. Assistive technology devices.

### Adolescence

- Got Transition: https://www.gottransition.org. Provides resources and guidance for transitions during adolescence.
- Couwenhoven T. *The boys' guide to growing up—choices and changes during puberty written for persons with intellectual disability*. Bethesda, MD: Woodbine House; 2012.
- Couwenhoven T. *The girls' guide to growing up—choices and changes during puberty written for persons with intellectual disability*. Bethesda, MD: Woodbine House; 2012.

### Across the Life Span

- Simon's Searchlight Foundation: https://www.simonssearchlight.org. An international research program with the goal of accelerating science and improving lives for people with rare genetic neurodevelopmental disorders.
- Family Voices: https://www.familyvoices.org/affiliates. Family-to-family health information centers that help families navigate systems needed by children with special health care needs.
- Parent to Parent USA: https://www.p2pusa.org/parents. Provides support to deal with the challenges of raising children with special health care needs.
- Parent Training and Information Centers: https://www.parentcenterhub.org. Resources in each state that inform, prepare, and assist families with navigation of the education system.
- ABLE ACT 2014: https://www.irs.gov/government-entities/federal-state-local-governments/able-accounts-tax-benefit-for-people-with-disabilities. Tax benefits for people with disabilities; allows states to create tax-advantage savings programs for eligible people with disabilities (designated beneficiaries) and can help pay for qualified disability expenses.
- Global Genes: https://www.globalgenes.org. Global Genes provides information, resources, and connections to all communities affected by rare diseases.
- Angel Aid: https://www.angelaidcares.org. Angel Aid connects families with other Rare Caregivers, helps them learn the tools of self-care, and allows them to be listened to without judgment.
- 16p11.2 Genetic Foundation: https://16pdel.org. A not-for-profit organization aiming to close the gap between cutting-edge research and individuals with 16p11.2 deletion syndrome.

### Competing Interest Statement

The authors have declared no competing interest.
